# PIV-Based Acoustic Pressure Measurements of a Single Bubble near the Elastic Boundary

**DOI:** 10.3390/mi11070637

**Published:** 2020-06-29

**Authors:** Qidong Yu, Zhicheng Xu, Jing Zhao, Mindi Zhang, Xiaojian Ma

**Affiliations:** 1 Department of Research and Development, China Academy of Launch Vehicle Technology, Beijing 100076, China; yuqidong124@126.com (Q.Y.); littlecz@163.com (Z.X.); sand810125@163.com (J.Z.); 2 School of Mechanical Engineering, Beijing Institute of Technology, Beijing 100081, China

**Keywords:** particle image velocimetry (PIV), single bubble, acoustic pressure, flow structure

## Abstract

The objective of this paper was to investigate acoustic pressure waves and the transient flow structure emitted from the single bubble near an elastic boundary based on the particle image velocimetry (PIV). A combination of an electric-spark bubble generator and PIV were used to measure the temporal bubble shapes, transient flow structure, as well as the mid-span deflection of an elastic boundary. Results are presented for three different initial positions near an elastic boundary, which were compared with results obtained using a rigid boundary. A formula relating velocity and pressure was proposed to calculate the acoustic pressure contours surrounding a bubble based on the velocity field of the transient flow structure obtained using PIV. The results show the bubbles near the elastic boundary presented a “mushroom” bubble and an inverted cone bubble. Based on the PIV-measured acoustic pressure contours, a significant pressure difference is found between the elastic boundary and the underside of the bubble, which contributed to the formation of the “mushroom” bubble and inverted cone bubble. Furthermore, the bubbles had opposite migration direction near rigid and elastic boundaries, respectively. In detail, the bubble was repelled away from the elastic boundary and the bubble was attracted by the rigid boundary. The resultant force made up of a Bjerknes force and buoyancy force dominated the migration direction of the bubble.

## 1. Introduction

The occurrence of cavitation bubbles is accompanied by many serious hazards, such as noise emission [1,2], material damage [3,4], and performance drops in hydraulic machinery [5,6]. The behaviors of bubble pulsation are significantly complex, including expansion, contraction, high-speed jet, mushroom shape, and opposite migration etc. [7,8,9,10]. It often involves the basic mechanical problems of the interaction of multiple physical fields, such as a bubble and a boundary, a bubble and an acoustic pressure wave, acoustic pressure wave and boundary, and acoustic pressure wave and acoustic pressure waves, which brings significant challenges to understand the behaviors of bubble pulsation and the evolution mechanism of acoustic pressure waves. Among them, the pressure wave, as the main energy transfer mechanism of bubbles in a liquid, is an important basis for revealing the mechanism of bubble pulsation [11,12,13,14].

When bubble is in the free field, the acoustic pressure wave released by the bubble pulsation will gradually spread to the far field; however, when the bubble is pulsating near the elastic boundary, the acoustic pressure wave released by the bubble will act on the bubble itself again through the boundary reflection, which has a complex impact on the pulsation shape and collapse characteristics of the bubble [15]. The reflection wave is coupled with the pressure wave released by the bubble at the moment, which further increases the complexity of the pressure distribution and bubble shapes [16]. Klaseboer et al. [17] employed the boundary element method (BEM) to investigate the bubble shape near the elastic boundary. They found the bubble present the “mushroom” shapes when initial position of the bubble inception is small. And then they proposed a possible explanation for the formation of “mushroom” bubble that “a certain amount of energy is stored during the deformation of the elastic boundary. Part of this energy is then released continuously a culminating when the bubble is near its maximum size”. Turangan et al. [18] also used BEM to investigate the bubble shape near the membrane and thought that “the elastic boundary repulses back towards the bubble and transfers its momentum to the bubble in a form of perturbation that propagates from the bottom to the top side of the bubble’s surface”. As for the bubble migration, Brujan et al. [19] used high-speed camera to record that the bubble is repelled by the polyacrylamide (PAA) boundary. They also put forward a hypothesis that the bubble is repelled by the elastic boundary. Based on the above literature, it is found that though “mushroom” bubble and its motion away from the elastic boundary have been paid attention for many years, they thought that both phenomena result from the release of the elastic potential energy of the boundary, which is compressed when bubble expands. The acoustic pressure released from the pulsating bubble near the elastic boundaries is also worthy investigated to reveal the fundamental mechanism of bubble dynamics. On the other hand, their formation mechanisms are all obtained based on speculation, which is worth further studying by experimental method and quantitative analysis.

High-speed camera is a common technology to record the temporal evolution of the bubble shapes, which has been widely reported in the works of Blake et al. [20], Wu et al. [21], Lauterborn et al. [22], Klaseboer et al. [23], Shima et al. [24] and Ma et al. [25]. Although the high-speed camera can capture a bubble’s instantaneous structures and outlines, it cannot present the flow structure and pressure field around the bubble. Kröninger et al. [26] investigated the velocity field in the vicinity of a laser-generated cavitation bubble by means of particle tracking velocimetry (PTV) and numerical simulation. They found that the accuracy of the PTV method is good agreement with assessed the experimental data with the flow field around the bubble. Vogel and Lauterborn [27] studied the flow field around bubbles during their collapse near a solid boundary by combining particle image velocimetry (PIV) and high-speed photography. They found that velocities could be determined within a range of from 2 m/s to 30 m/s and within a field of 10 × 10 mm^2^. A temporal resolution of 10 kHz and a spatial resolution of higher than 2 points/mm^2^ have been achieved. Besides, Shangguan et al. [28] also applied time-resolved PIV to investigate the flow field during the bubble oscillation near a soft boundary. In the past years, the availability of PIV technologies to acquire high-resolution measurements has motivated its application for the multiphase flow dynamics to obtain the measurements of fluid pressure distribution [29,30,31]. Auteri et al. [32] proposed a method to calculate the pressure distribution using PIV. This methodology consists of two steps: (i) the first step generates the Neumann boundary conditions by solving inversely the Naiver-Stokes equation; (ii) the second one involves a numerical solution of the pressure Poisson equation. The detailed information about calculated pressure by PIV can be reviewed in the works of Morris [33] and Kat et al. [34]. But the pressure distribution using PIV has not been applied for the field of single bubble dynamics near the elastic boundary. A new method, based on the weakly compressible effect of the liquid surrounding a bubble, is proposed to build the relationship between the pressure and velocity in this work to investigate the flow structure around a bubble.

The main research content of this paper is organized as follows. In Section 2, the experiment is illustrated including bubble generation, boundary information, high-speed photographing and time-resolution PIV. In Section 3, a new method is proposed to calculate the pressure distribution around a bubble by PIV experimental data, which originates from the state of equation and compressible mass conservation equation. In Section 4, we study two groups of bubble dynamics near the elastic boundary. The first one is to investigate the “mushroom” bubbles and its mechanism, while the second one is to study the opposite bubble migration as compared with the case of the rigid boundary. In Section 5, this new study is summarized and the key outcomes are identified.

## 2. Experimental Method

### 2.1. Bubble Generation

**Figure 1:**
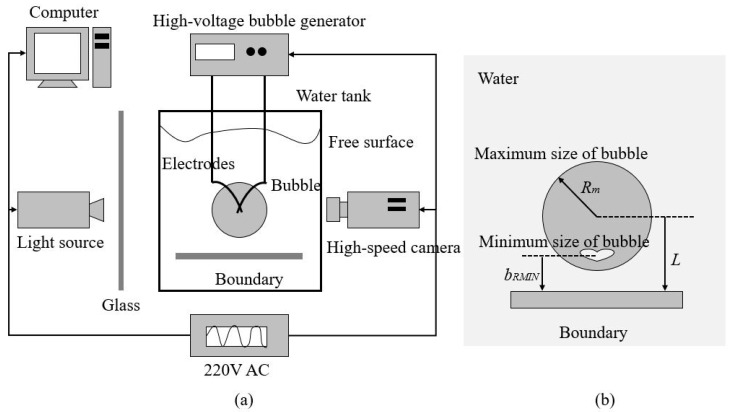
(**a**) Schematic diagram of the experimental equipment, including the high-speed camera system and bubble generator; (**b**) details regarding the position of a bubble relative to the plate in space.

The experiment is carried out in a cuboid-shaped water tank with a height of 1000 mm and a square bottom with lengths of 500 mm, as shown schematically in Figure 1a. To achieve better photography and illumination, the water tank is made of transparent glass and is partially filled with sufficiently degassed water. The temperature of water in the tank is maintained at 298 K. The bubbles are generated via Joule heating at the connecting point of the electrodes resulting from the discharge of a 6600 μF charge to 800 V, as shown in Figure 1a. Upon discharge, copper electrodes, with a 0.3 mm diameter, evaporate the water at the connecting point, emit an extremely high temperature, and create a bubble with rapid expansion, namely, an electric spark-induced bubble. To quantitatively describe a bubble in an infinite fluid, the maximum radius of the bubble is defined as

(1) $$R_{m}=\sqrt{A/\pi }$$

where *A* is the maximum area of the bubble on the screen. The centre of the initial bubble is shown to always be located at the connecting point. Therefore, it is possible to precisely control the spatial location of the initial bubble. In the present experiment, bubbles are generated over the boundary; detailed information regarding the position of a bubble relative to the plate is shown in Figure 1b. The normalized initial position of the bubble is defined as

(2) $$\gamma =\frac{L}{R_{m}}$$

where *L* is the distance from the bubble centre to the boundary. In order to measure the collapse position of bubble, a normalized variable is defined as

(3) $$\beta =\frac{b_{BRIM}}{L}$$

where *b_RMIN_* is the distance between boundary and bubble centre of minimum size in Figure 1b.

### 2.2. Boundary Information

In the experiment, two kinds of materials are investigated, namely, carbon-fibre composite and standard aluminium samples. They are processed with the same length of 120.0 mm and width of 80.0 mm, but not the height. In order to make the carbon fiber composite to be easily yielding, it is machined with the height of 0.5 mm, which is considered as an elastic boundary. And the aluminium sample is about 3.0 mm in height, which is considered as a rigid boundary referred from classic work of Brujan et al. [13,14] and Hung et al. [35]. The stiffness coefficient *k* can be used for judging the rigidity of the carbon-fiber composite and aluminum sample. Stiffness coefficient *k* of composite sample is about 1.4 N/mm, while *k* of aluminum sample is about 1472 N/mm. It can be seen that the stiffness coefficient of aluminum sample is enough larger to be treated as rigid boundary, as compared with that of carbon-fibre composite. The elastic properties of the elastic boundary samples are strained by a universal test machine. The boundary is quantified by determining the stress-strain relation and calculating the elastic modulus, *E*, as the slope of the stress-strain curves. Under compression, the boundary sample brakes at a stress value of 2750 N. The other important information including size, elastic modulus, and density can be founded in Table 1. In the experiment, the two shorter ends of the plates are clamped, as shown in Figure 1b.

### 2.3. High-Speed Photography

The temporal evolution of the bubble dynamics is recorded using a high-speed camera (Phantom V12.1) operating in 25,000 frames per second (fps). To ensure the sharpness of the bubble outline and its inner structure, the exposure time of each frame is set to 30 μs. Diffusive illumination is provided by a continuous light source at one side of the water tank, namely, the side opposite the high-speed camera, as shown in Figure 1a. To achieve a better light distribution around a bubble, a piece of glass with thickness of 3 mm is placed between the water tank and light source. The high-speed camera and copper electrodes are almost synchronously triggered, with the maximum error in delay time for both being approximately 0.067 ms, which is small enough to be neglected compared to the duration of bubble oscillation (about 4 ms).

### 2.4. PIV System

**Figure 2:**
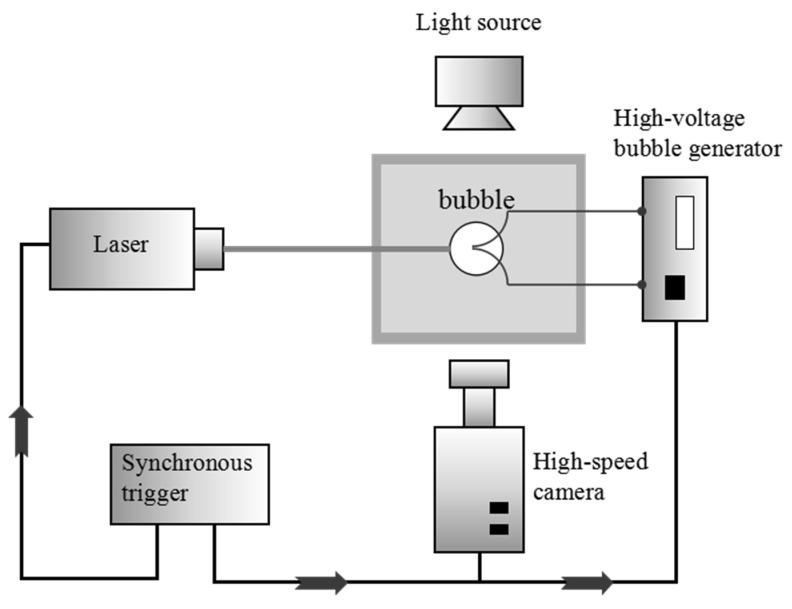
Schematic drawings of the bubble generator system with a top view synchronized with laser and high-speed camera.

A 2D time-resolved PIV system composed of RayPower 5000 Laser and SpeedSence M310 camera is used to measure the flowing fields around a bubble and its layout is shown in Figure 2. The light source of RayPower Laser is a gas cooled laser head. In this study, the RayPower Laser is used in continuous mode to form a continuous light sheet in order to illuminate the fluid field around a bubble and be further record by the high-speed camera with a resolution of 12 bits, 800 × 512 pixels^2^. The evaluation of the vector fields from the PIV images is performed with the commercial PIV software and an overview of the parameters is presented in Table 2.

The fluid information around the surface of the boundary is so important to understand the characteristics of Fluid Structure Interaction for bubble near an elastic boundary that an optical filter and fluorescent paint are applied to block reflections of the light-sheet from the elastic and rigid boundaries. The velocity fields around a bubble can be captured using the hollow glass micro-spheres with diameter of 50 μm as “tracer particles”. The commercial PIV-software, named Dynamic Studio is used to process the velocity vector fields with the interrogation areas of 32 × 32 pixels and 50% overlap in general. The uncertainty in the velocity measurement is related to uncertainty in the spatial and temporal measurements of the PIV system. The light source of a laser is used in continuous mode to form a continuous light sheet. The uncertainty in the measured time interval was less than 0.2%. The uncertainty in the spatial data is largely related to the magnification of image and the size of interrogation areas. A variety of criteria are used to validate the individual vectors and the uncertainty is limited to approximately 5.2% during the vector validation.

## 3. Acoustic Pressure Prediction Method

**Figure 3:**
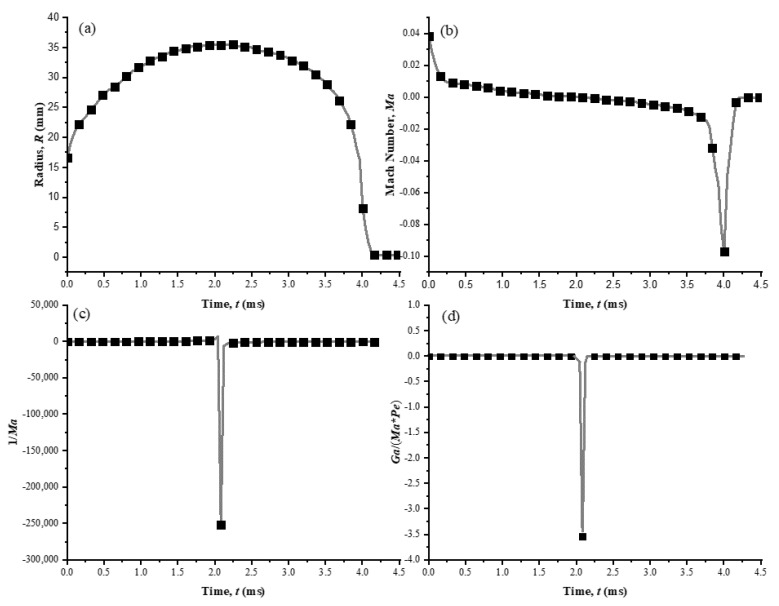
The companions of (**a**) *R*, (**b**) *Ma*, (**c**) 1/*Ma*, and (**d**) *Ga*/(Ma*Pe) for the case of bubble dynamics near the rigid boundary, where *R* is the instantaneous radius of the bubble; *Ma* is the Mach number; *Pe* the Peclet number; and *Ga* the Gay-Lussac number [37].

In this part, a formula about relationship between pressure and velocity will be introduced to get the pressure distribution around a bubble. And the non-dimensional equation for pressure can be defined as [36,37]

(4) $$\frac{\partial p^{*}}{\partial t^{*}}=-\frac{1}{Ma}\nabla \cdot u^{*}-Ma\cdot u^{*}\cdot \nabla p^{*}+\frac{1}{Ma}\frac{1}{Pe}Ga\nabla^{2}T^{*}$$

where *p* is pressure, *t* is time, ***u*** is velocity, *Ma* is the Mach number, *Pe* is the Peclet number *Ga* is the Gay-Lussac number, and superscript * represents the normalized variable superscript * represents the normalized variable. And the dimensionless parameters in Equation (4) are defined as follows:

(5) $$p^{*}=\frac{p}{\rho V_{0}^{2}};u^{*}=\frac{u}{V_{0}};t^{*}=\frac{tV_{0}}{H};T^{*}=\frac{T}{T_{0}}$$

where *ρ* is density, *V*_0_ is a reference velocity, *T*_0_ is a reference temperature, *H* is a characteristic length. To further simplify the equation about the pressure, the three terms of the right hand in Equation (4) are considered in detail to get the expression suitable for the case of bubble dynamics near the rigid boundary. Figure 3 shows the companions of *R*, *Ma*, 1/*Ma*, and *Ga*/(Ma*Pe) for the case of single bubble near the rigid boundary. As observed, *R* is the top margin of the bubble obtained from the experimental observation. Furthermore, compared with the absolute value of 1/*Ma*, the values of *Ma* and *Ga*/(Ma*Pe) are so small in magnitude and both can be neglected in the Equation (4). Therefore, the final formation of the mass conservation can be expressed as,

(6) $$\frac{\partial p^{*}}{\partial t^{*}}=-\frac{1}{Ma}\nabla \cdot u^{*}$$

And it is written in real variables again as,

(7) $$\frac{\partial p}{\partial t}=-\frac{1}{\chi_{T}}\nabla \cdot u$$

where *χ_T_* = 0.444 × 10^−9^ P^−1^ for water. To calculate the pressure with the velocity data obtained by PIV easily, Equation (7) is rewrite with the difference form

(8) $$p^{n+1}=p^{n}-\frac{\Delta t}{\chi_{T}}\nabla \cdot u^{n+1}$$

where Δ*t* is the time interval of PIV data acquisition; *n* is the calculated step. According to our previous work and investigation [38], bubbles near the boundary are deemed as axisymmetric shapes, and hence the flow can be processed in cylindrical coordinates. Therefore, ∇⋅u in Equation (8) can be expressed as

(9) $$\nabla \cdot u=\frac{1}{r}\frac{\partial }{\partial r}\left(ru_{r}\right)+\frac{\partial }{\partial z}\left(u_{z}\right)+\frac{1}{r}\frac{\partial }{\partial \theta }\left(ru_{\theta }\right)$$

where *r* is distance to the vertical cylindrical axis, *z* is cylindrical height, and *θ* is angle. In order to simplify the calculation, 3D flow structure in cylindrical coordinates can be treated as 2D plots by neglecting the third term of right hand in Equation (8).

## 4. Results and Discussions

Three different initial positions of the bubble inception are investigated near elastic and rigid boundaries, namely *γ* = 0.81, 1.20 and 1.68. We study the “mushroom” bubbles in the first group with *γ* = 0.81 and 1.20 for elastic boundary. As for the second group, the companion of two cases with *γ* = 1.68 is displayed near the elastic and rigid boundaries to illuminate the mechanism of its opposite bubble migration.

### 4.1. The Formation of “Mushroom” Bubble

#### 4.1.1. The Case of γ = 0.81

**Figure 4:**
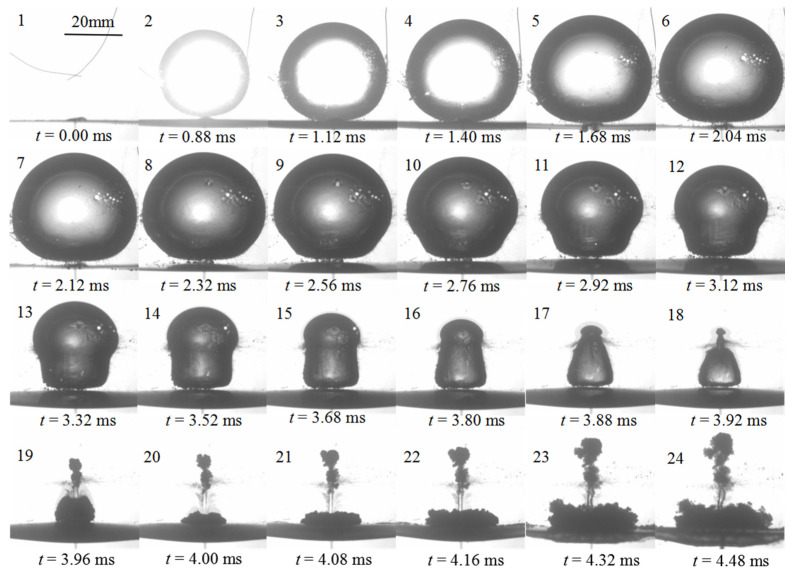
Temporal evolution of bubble shapes for *γ* = 0.81 and *R_m_* = 19.4 mm.

Figure 4 shows the high-speed photographs of “mushroom” bubble shapes closely near the elastic boundary for *γ* = 0.81 and *R_m_* = 19.4 mm. There exist three stages of a bubble shape, namely expansion, shrink, and splitting ones. Frames 1–6 display the expansion stage of the bubble that a bubble is initiated at *L* = 15.7 mm away from the flat elastic boundary when *t* = 0.00 ms and then the bubble volume sharply expands to the maximum size from *t* = 0.88 ms to *t* = 2.04 ms. Due to the restrictions of the elastic boundary, the bottom margin of the bubble becomes flattened in the direction parallel to the boundary, which is obviously observed at frame 6. Frames 7–18 give the shrink stage of the bubble. During the continuous reduction in volume, the bubble presents three distinct features: (i) The bottom margin of bubble is flattened and is almost in contact with the elastic boundary, leaving a liquid veneer in between; (ii) the curvature of the top margin of the bubble is almost invariable, though the bubble volume is decreasing; and (iii) a disturbance begins from the bottom margin of the bubble, then moves along the bubble interface upwards, and finally results in the splitting off of a small bubble at the top, which presents a complete formation process of “mushroom” bubbles. Frames 19–24 illustrate the formation of splitting process. As observed, the bubble is torn into two segments that upper one is counter jet and lower one is the annular flow. The counter jet moves upwards with the mean velocity of 7 m/s, and the annular flow impinges the elastic wall.

**Figure 5:**
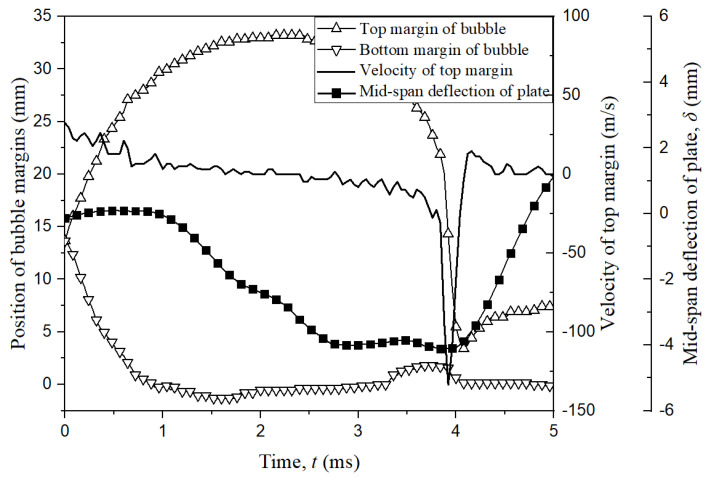
The comparison of the temporal evolution of two bubble margins, velocity of top margin, and mid-span displacement of elastic boundary for *γ* = 0.81 and *R_m_* = 19.4 mm.

The problem of bubble and elastic boundary is a class case of Fluid-Structure Interaction; therefore, the dynamic response of the elastic boundary is essential to get comprehensively and further understanding in this phenomenon. Figure 5 shows the comparison of the temporal evolution of two bubble margins, velocity of top margin and mid-span displacement *δ* of the elastic boundary. The solid line is time history of the mid-span deflection of the boundary. Besides, the hollow upward triangles indicate the temporal evolution of top margin of bubble, while the downward triangles represent the bottom margin of the bubble. It is noted that the negative value of *δ* represents the boundary moves downwards and positive one indicates the boundary moves upwards, which are obtained from the high-speed photography. As observed, the bubble expansion results in the compression of the elastic boundary during *t* = 0–2.04 ms. Furthermore, *δ* continues to decrease under the function of the vibration force to the minimum value of *δ =* −4 mm when *t* = 3.9 ms. Currently, the bubble almost stays the minimum volume, the velocity of top margin increases to 130 m/s, and the boundary begins to repulse back upwards under the elastic potential energy. During this process, the mid-span deflection is always a negative value (*δ* < 0), indicating the boundary presents a concave shape during the bubble oscillation. And the maximum velocity of the boundary vibration is about 5 m/s.

**Figure 6:**
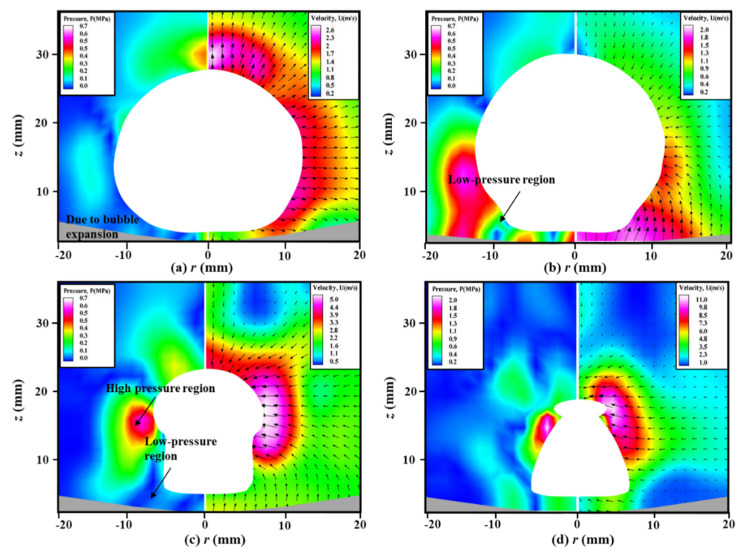
The measured velocity fields (right) and calculated pressure contours (left) around a bubble near the elastic boundary for *γ* = 0.81 and *R_m_* = 19.4 mm at typical time of (**a**) *t* = 0.88 ms, (**b**) *t* = 2.92 ms, (**c**) *t* = 3.68 ms and (**d**) *t* = 3.88 ms.

To further investigate the formation mechanism of the “mushroom” bubble, Figure 6 shows the measured velocity field of the transient flow structure and calculated pressure contours around a bubble near the elastic boundary, which is illustrated in Figure 4. The off-white areas represent the bubble outline, which are masked during the data processing by PIV software. The gray areas on the bottom of each image are the deformable boundary, which are depicted quantificationally according to Figure 5. As observed at *t* = 0.88 ms, the bubble is in the expansion stage, resulting in a relatively high-pressure region around the bubble surface, as shown in Figure 6a. The acoustic pressure difference pushes the velocity vectors to migrate outwards from the bubble surface. When *t* = 2.92 ms as shown in Figure 6b, a pressure difference between high-pressure region and low-pressure region is found between the boundary and the underside of the bubble, and the position of its center is about *z* = 10 mm. The velocity vectors at the region of *z* < 20 mm move from elastic boundary to the disturbance of the bubble surface, while that at the region of *z* > 20 mm move towards the surface of bubble from the finite liquid. The phenomenon indicates the deformation of the elastic boundary contributes to the formation of high-pressure region and migration of the velocity vectors. At *t* = 3.68 ms of Figure 6c, the area of the high-pressure region decreases evidently and its center moves upwards to about *z* = 15 mm, but still locates at the disturbance of the bubble surface. The majority of the velocity vectors direct to the disturbance of the bubble. When time goes to *t* = 3.88 ms as shown in Figure 6d, the high-pressure region locates at the splitting point of the topmost part of the bubble, the low-pressure region appears at bottom of the bubble and the direction of the vectors are tangent to the surface of the bubble, resulting in the splitting of the bubble.

#### 4.1.2. The Case of γ = 1.20

**Figure 7:**
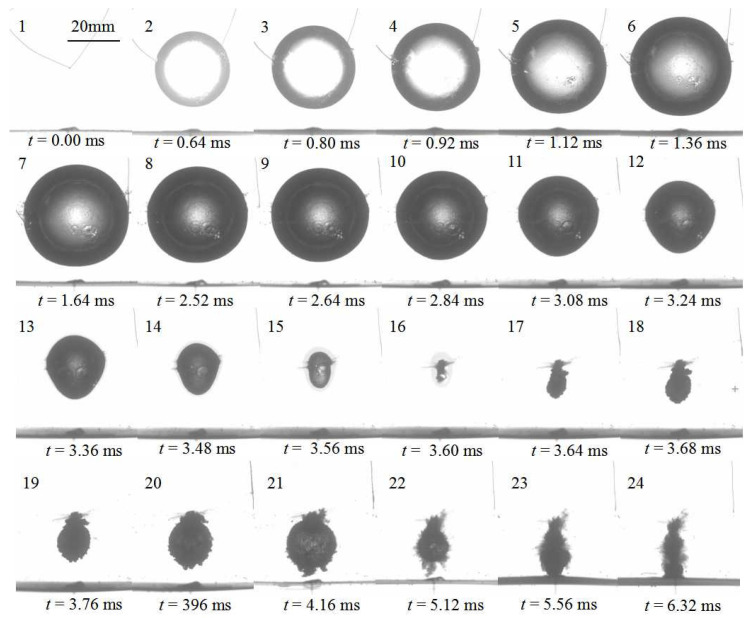
Temporal evolution of bubble shapes for *γ* = 1.20 and *R_m_* = 20.0 mm.

**Figure 8:**
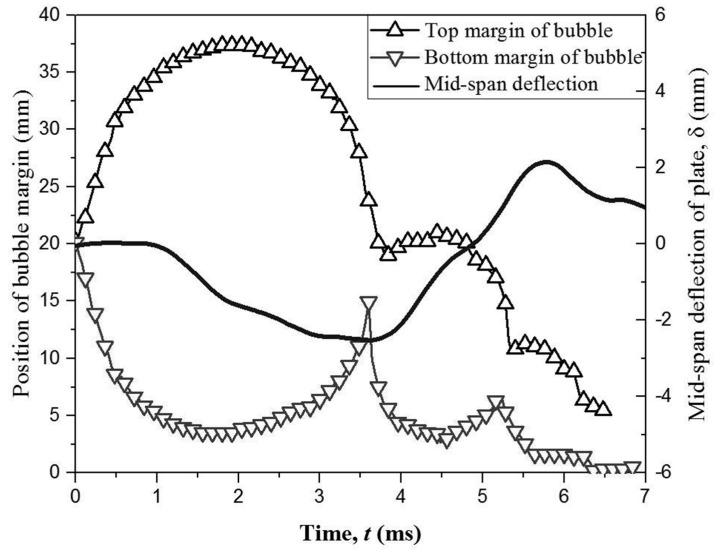
The comparison of the temporal evolution of two bubble margins and mid-span displacement of elastic boundaries for *γ* = 1.20 and *R_m_* = 20.0 mm.

The initial position of bubble inception increases slightly to *γ* = 1.20. Figure 7 shows the high-speed photographs of bubble shapes for *γ* = 1.20 and *R_m_* = 20.0 mm. In the expansion stage during *t* = 0–1.64 ms, the significant difference is that the bubble does not touch the elastic boundary when reaching the maximum volume, compared with the case of *γ* = 0.81. During the shrink stage *t* = 2.52 −3.60 ms, the top margin of the bubble remains a constant curvature, but the bottom margin becomes more and more sharp, due to the disturbance along the bubble surface from the bottom to the top. It is evident that spherical bubble transfers into an inverted cone shape, such as frame 12. During the rebound stage *t* = 3.64–6.32 ms, the bubble begins to expand again with spherical shape from the minimum volume. When *t* = 5.56 ms, the bottom margin of the bubble touches the elastic boundary. To further demonstrate the relationship between the bubble shape and boundary deformation, Figure 8 shows the comparison of the temporal evolution of two bubble margins and mid-span displacement of elastic boundaries for *γ* = 1.20 and *R_m_* = 20.0 mm. It is found that the mid-span deflection of the elastic boundary reaches to the minimum value of *δ* = −2.4 mm when bubble is in the minimum value (*t* = 3.60 ms). After that, the mid-span deflection begins to increase under the effect of elastic potential energy of the elastic boundary. Until *t* = 5.00 ms, the mid-span deflection reaches to the equilibrium position of *δ* = 0. Due to the inertial force of the boundary, the mid-span deflection continues to increase until the bubble touches the boundary at *t* = 5.56 ms.

**Figure 9:**
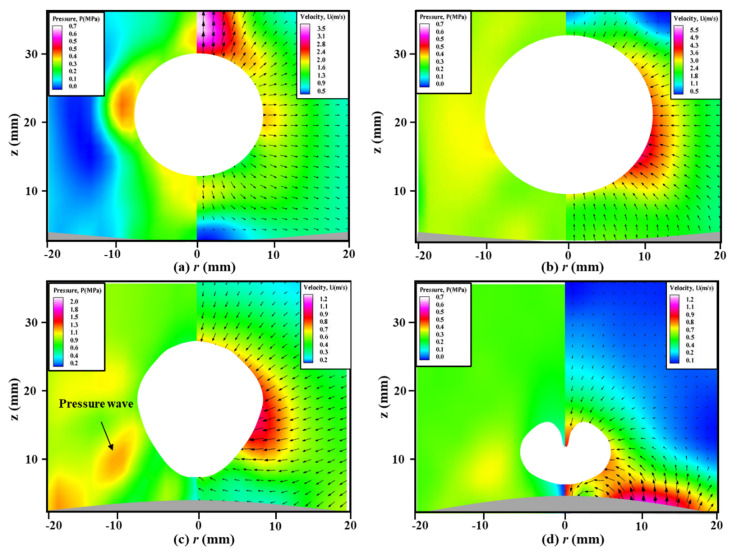
The measured velocity fields (right) and calculated pressure contours (left) around a bubble near the elastic boundary for *γ* = 1.20 and *R_m_* = 20.0 mm at typical time of (**a**) *t* = 0.92 ms, (**b**) *t* = 3.08 ms, (**c**) *t* = 3.36 ms and (**d**) *t* = 4.16 ms.

To further reveal the flow structure around this bubble, Figure 9 shows the measured velocity fields of the transient flow structure (right) and calculated pressure contours (left) around the bubble near the elastic boundary for *γ* = 1.20 and *R_m_* = 20.0 mm at typical time of *t* = 0.92 ms, 3.08 ms, 3.36 ms and 4.16 ms. As observed at Figure 9c, a high-pressure region is found between the elastic boundary and the underside of the bubble, which pushes the side margin of the bubble with high velocity. It concludes that the pressure difference between the high-pressure region and low-pressure region contributes to the formation of this disturbance along the bubble surface. Because the low-pressure region delays contraction of the bubble bottom margin, while the high-pressure region accelerates the contraction of the bubble top margin. As shown in Figure 9d, this high-pressure region still locates at the side of the sub-bubble.

**Figure 10:**
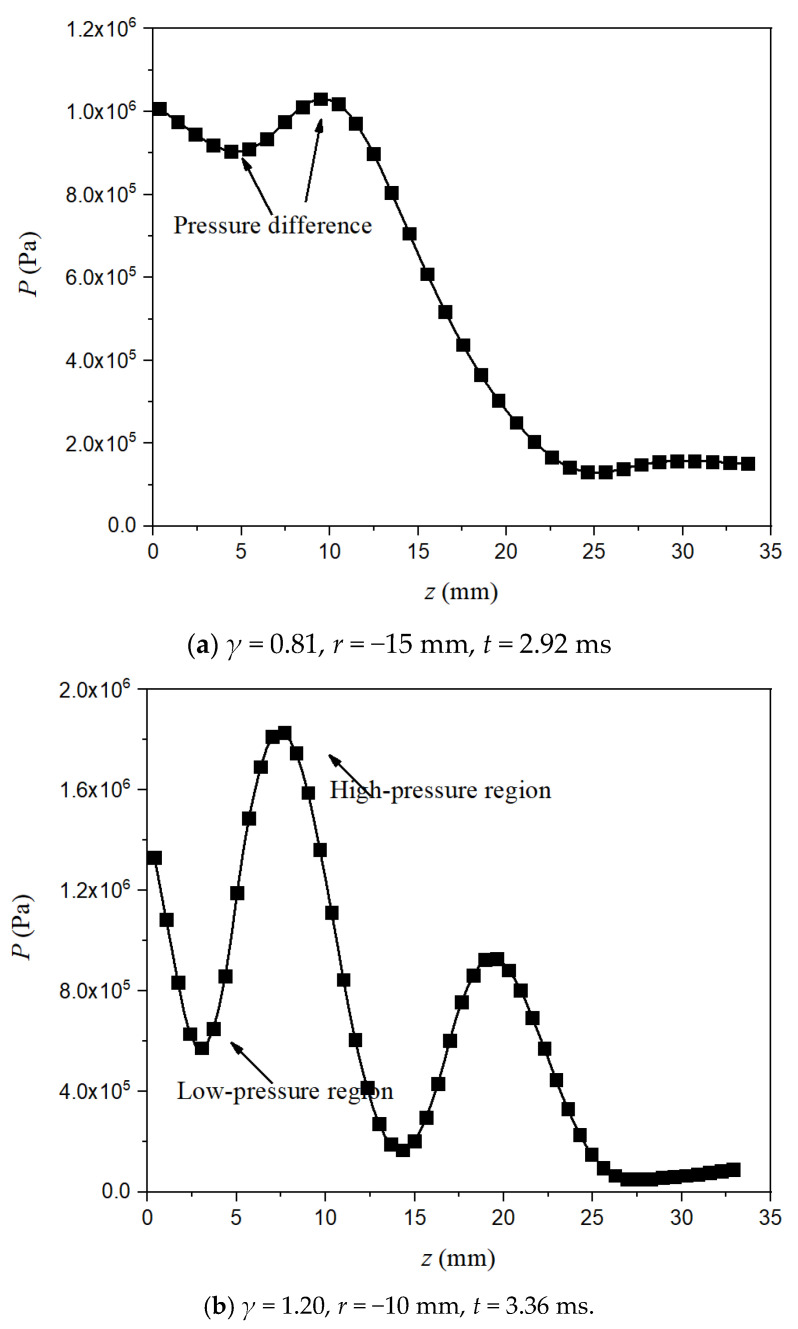
The comparisons of the measured high-pressure region for different conditions. The experimental value is obtained along the vertical axis, when γ = 0.81, *r* = −15 mm and *t* = 2.92 ms for image (**a**); and γ = 1.20, *r* = −10 mm and *t* = 3.36 ms for image (**b**).

In order to quantitatively investigate the high-pressure region, Figure 10 shows the comparison of the PIV-measured high-pressure region for γ = 0.81 and γ = 1.20, respectively. As observed, there is only one pressure peak for case γ = 0.81, while there are two pressure peaks for case γ = 1.20. It is found that the pressure difference around the underside of the bubble is the reason to cause the disturbance of the bubble surface and the formation of “mushroom” bubble (or inverted cone bubble when initial position is large). That’s because the pressure difference causes the distinctly different velocity of bubble margin contraction.

### 4.2. The Bubble Migration

**Figure 11:**
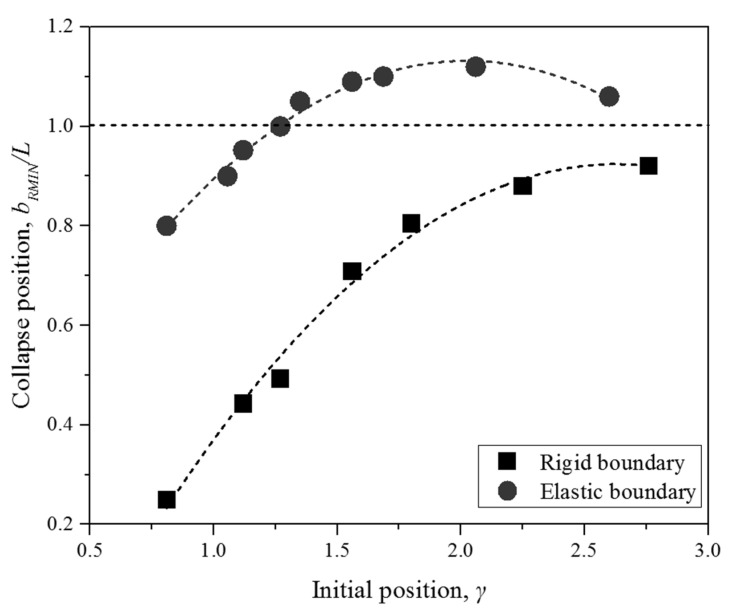
The comparison of bubble initial position and collapse position for elastic and rigid boundaries.

The bubble migration is one of most important characteristics of a bubble. The previous works focus on the bubble migration away from the boundaries aiming at preventing cavitation at the surface of the fluid machinery. Figure 11 shows the comparison of bubble initial position *γ* and collapse position *b_RMIN_/L* near elastic and rigid boundaries, where *b_RMIN_* is the height above the wall at which the north and south poles of the bubble meet each other in Figure 1. It is noted that collapse position *b_RMIN_/L* > 1 indicates bubble moves away from the boundary, *b_RMIN_/L* < 1 represents bubble migrates closely to the boundary, and *b_RMIN_/L* = 1 is that the center of bubble keep motionless. As observed, when the initial position *γ* > 1.25, the collapse position *b_RMIN_/L* is always larger than 1 for the elastic boundary, while *b_RMIN_/L* is always smaller than 1 for the rigid boundary. That indicates the bubble always moves away from the elastic boundary, and migrates towards the rigid one when *γ* > 1.25.

**Figure 12:**
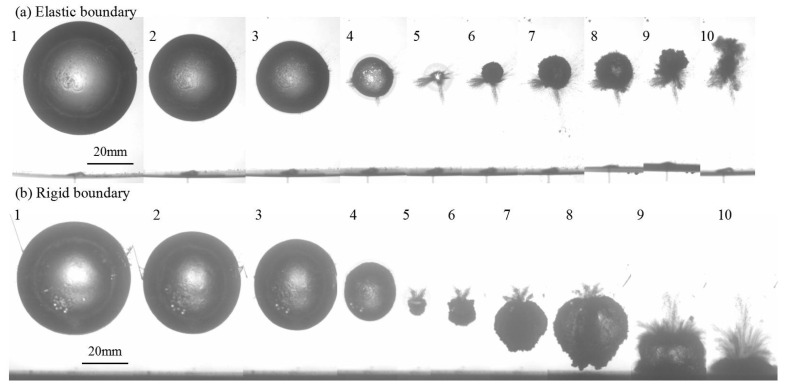
The bubble migration near the elastic and rigid boundaries for *γ* = 1.68 and *R_m_* = 20.0 mm.

To further demonstrate the detail bubble shapes, Figure 12 shows the temporal evolution of typical bubble shapes near the elastic and rigid boundaries for *γ* = 1.68 and *R_m_* = 20.0 mm. As observed, two bubbles both oscillate at a far distance from the elastic and rigid boundaries, the Bjerknes force between the bubbles and boundary tends to be weak, resulting in a nearly spherical bubble oscillation for the first period. As shown in Figure 12a, once reaching at the minimum volume at frame 5, the bubble near the elastic boundary has a bulge toward the interior of a bubble at the bottom of the bubble surface, and it moves upwards evidently with several oscillation cycles. As for the bubble near the rigid boundary shown in Figure 12b, the bubble has a sunken at the top of the bubble surface after frame 5, and then it is attracted by rigid boundary and fiercely impacts on the rigid wall with two period of bubble oscillation.

**Figure 13:**
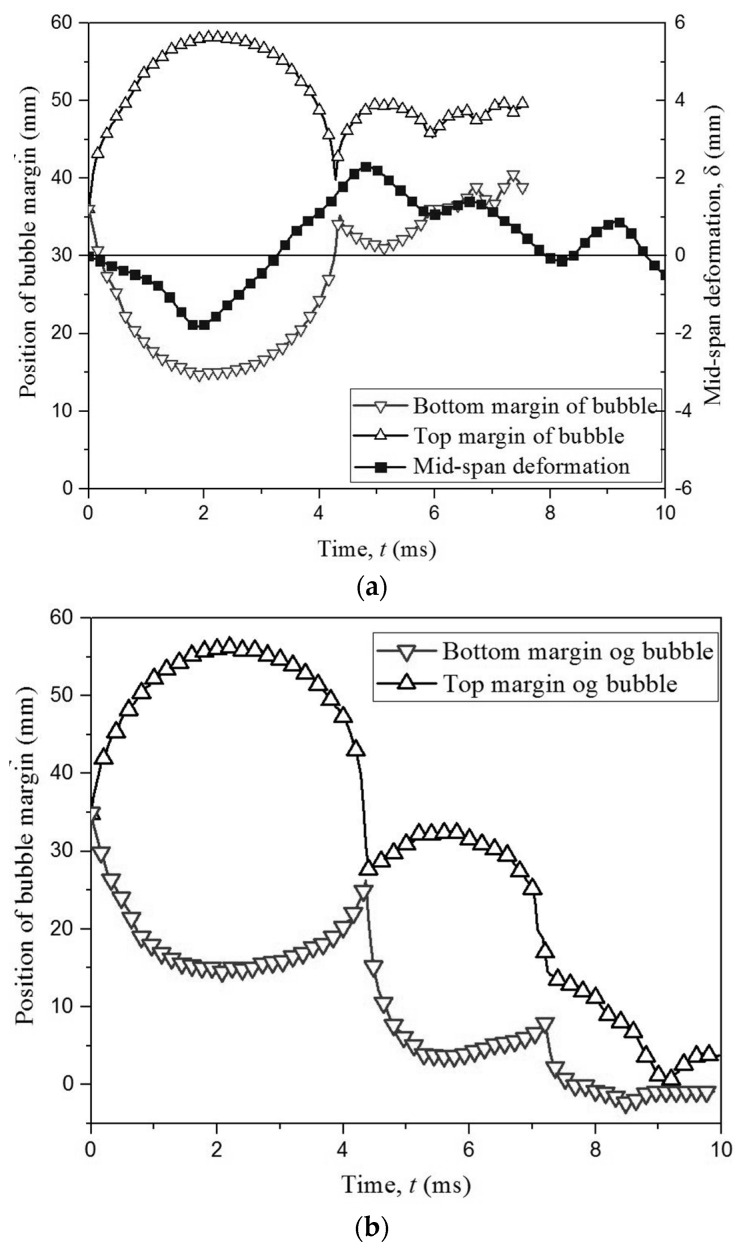
(**a**) The comparison of the temporal evolution of two bubble margins and mid-span displacement of elastic boundaries for *γ* = 1.68 and *R_m_* = 21.0 mm [39]; (**b**) the temporal evolution of two bubble margins near the rigid boundary for *γ* = 1.68 and *R_m_* = 21.0 mm.

To further demonstrable the relationship between the bubble motion and the boundary vibration, Figure 13a shows the comparison of the temporal evolution of two bubble margins and mid-span displacement of elastic boundaries for *γ* = 1.68 and *R_m_* = 20.0 mm, and its temporal shapes are shown in Figure 12a. When the bubble expands from *t* = 0 ms to *t* = 2.5 ms, the elastic boundary is compressed by the bubble, and its mid-span deflection keep decreasing trend to the minimum value of *δ* = −2.0 mm at *t* = 2.5 ms. After that, the bubble begins to shrink and the elastic boundary also pushes back towards the equilibrium state. At *t* = 4.0 ms, the elastic boundary reaches to the equilibrium state, but it still has an upwards motion due to vibration force. When the bubble reaches to the minimum volume at *t* = 4.36 ms, the min-span deflection presents a positive value of *δ* = 0.5 mm. As for the bubble near the rigid boundary, the mid-span deflection of the boundary is neglected due to its large stiffness, as shown in Figure 13b.

**Figure 14:**
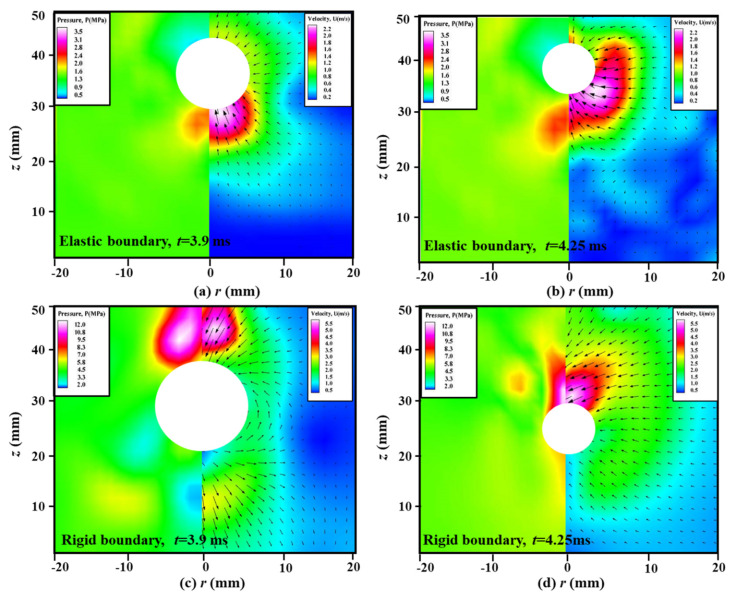
The measured velocity fields (right) and calculated pressure contours (left) around a shrinking bubble: (**a**) and (**b**) near the elastic boundary for time *t* = 3.9 ms and 4.25 ms; (**c**) and (**d**) near the rigid boundary for time *t* = 3.9 ms and 4.25 ms. The high-pressure region is caused by the Bjerknes force, buoyancy and vibration force for elastic boundary, while it is only caused by Bjerknes force and buoyancy for rigid boundary.

Figure 14 shows the PIV-measured velocity fields and calculated pressure contours around a shrinking bubble, where Figure 14a,b are from the case of elastic boundary for time *t* = 3.90 and 4.25 ms, and Figure 14c,d are from the case of rigid boundary for time *t* = 3.90 and 4.25 ms. Due to the similarity with Figure 9, the expansion stages in this figure are neglected, and the shrink stages of bubbles become the focus of attention in this part. It can be seen that there exists a high-pressure region at the bottom of the bubble surface near the elastic boundary with the maximum value of 3.5 MPa, while it appears at the top of the bubble surface near the rigid boundary with the maximum value of 12.0 MPa. Although two bubbles have the same initial position and maximum radius, the flow structures around them are obviously different, even opposite. It is well known that a bubble near the rigid boundary is always affected by a pair of opposing forces, namely, the buoyancy and Bjerknes force reported by Blake et al. [40]. As for the elastic boundary case, the high-pressure region appears at the bottom of the bubble, indicating that Bjerknes force is smaller than the buoyancy force. As for the rigid boundary case, the high-pressure region appears at the top of the bubble, indicating that Bjerknes force is much larger than the buoyancy force. Therefore, the resultant force of Bjerknes force and buoyancy force dominate the migration direction of the bubble.

## 5. Conclusions

Experimental studies are presented for bubble dynamics near the elastic boundary for different initial position. High-speed videos of the evolution of the bubble patterns mid-span deflection of the elastic boundary, and PIV measurements of velocity and pressure fields are used to investigate the flow structure around the bubble. The primary findings include:

(1) A formula between pressure and velocity is proposed to calculate acoustic pressure contours surrounding a bubble based on the velocity field obtained by PIV. To simplify the equation about the pressure, the three terms about Mach number are considered in detail to get the expression suitable for the case of bubble dynamics. And the relatively smaller terms in quantity are neglected in the formula.

(2) The bubbles near the elastic boundary presents “mushroom” bubble and inverted cone bubble. Based on the PIV-measured acoustic pressure contours, a pressure difference region is found between the elastic boundary and underside of the bubble, which contributes to the formation of the “mushroom” bubble and inverted cone bubble.

(3) The bubbles have opposite migration direction near rigid boundary and elastic boundary, respectively. In details, the bubble is repelled away from the elastic boundary and the bubble is attracted by the rigid boundary. The resultant force of Bjerknes force and buoyancy force dominate the migration direction of the bubble.

Regarding the future work, PIV-based acoustic pressure measurements of a single bubble should be further developed to get more accurate pressure and velocity data, which will provide the method of experimental verifications in velocity and pressure fields and significantly promote the development of the CFD simulation in treating the problem of Fluid-Structure Interaction.

## Figures and Tables

**Table 1:** The detailed parameters about the boundary.

Items	Materials	Length *a* (mm)	Width *b* (mm)	Height *c* (mm)	Elastic Modulus *E* (GPa)	Density *ρ* (kg/m^3^)
Elastic boundary	Carbon fiber composite	120.0	80.0	0.5	66.2	1286
Rigid boundary	Aluminum	120.0	80.0	3.0	70.0	2700
Values of elastic modulus, density of the boundary samples at 298 K						

**Table 2:** Overview of PIV parameters.

Seeding	Type	Hollow glass micro sphere	
	Diameter	50	μm
	Type	Gas bubbles	
Light sheet	Laser type	RayPower 5000	
	Laser power	5	W
	Wave length	532	nm
	Laser light mode	Continuous mode	
Camera	Type	SpeedSence M310	
	Resolution	800 × 512	Pixels^2^
	Interframe time	0.25	ms
	Pixel size	20	μm
	Pixel depth	12	bit
	Memory	6	GB
PIV analysis	Interrogation area	32 × 32	Pixels^2^
	Overlap IA	50%	
